# Influence Reclaimed Asphalt Shingles on the Physicochemical and Rheological Properties of Road Bitumen

**DOI:** 10.3390/ma18184291

**Published:** 2025-09-12

**Authors:** Krzysztof Kołodziej, Szymon Malinowski, Wojciech Franus

**Affiliations:** 1 Faculty of Civil and Environmental Engineering and Architecture, Rzeszow University of Technology, Powstancow Warszawy 12 Street, 35-959 Rzeszow, Poland; 2 Faculty of Civil Engineering and Architecture, Lublin University of Technology, Nadbystrzycka 40, 20-618 Lublin, Poland; s.malinowski@pollub.pl

**Keywords:** manufactured asphalt shingles, tear-off asphalt shingles, asphalt bitumen performance, recycling

## Abstract

The article presents the results of a study on 50/70 paving-grade bitumen modified with the bitumen recovered from two types of asphalt shingles: post-consumer asphalt shingles (TOAS) and manufacturing waste asphalt shingles (MWAS) at three dosage levels (15%, 30%, and 45% *w*/*w*). The evaluation included the basic properties of bitumen—its penetration and softening point—as well as rheological properties, such as its viscosity, fatigue life determined by the LAS method, and rutting resistance assessed using the MSCR test and FTIR analysis. In both cases, the results showed that an increase in the stiffness of the base bitumen was observed. An improvement in rutting resistance was also recorded, as evidenced by the reduction of J_nr3.2_, along with an increase in fatigue life. A stronger stiffening effect was found in the case of the TOAS-derived bitumen, which is related to aging processes occurring during its service life. This suggests that the maximum allowable content of the additive should depend on the source of the reclaimed asphalt shingles, with MWAS being applicable in larger amounts without excessive deterioration of bitumen performance. The key contribution of this study is the demonstration that the MWAS and TOAS additives cannot be treated equally, as each affects the base bitumen differently.

## 1. Introduction

Asphalt shingles are one of the most widely used roofing covers. Depending on the type, they contain approximately 20 to 35% bitumen, making them a valuable waste material that can be reused in asphalt mixtures [1]. This is particularly important in the context of a circular economy, which aims to minimize the use of non-sustainable, virgin, carbon-intensive, non-renewable materials and waste generation. Incorporation of asphalt shingles into road construction materials not only reduces the cost of raw materials, but also lowers the expenses associated with its disposal [2].

On the basis of their origin, the waste material from asphalt shingles (RAS) is classified into tear-off asphalt shingles (TOAS, post-consumer) and manufactured waste asphalt shingles (MWAS, post-manufacture) [3,4]. In the United States, MWAS is predominantly used, due to legal restrictions on the use of materials containing asbestos. However, it is no longer used in the production of new asphalt shingles, and this restriction is being questioned by some researchers as unfounded [3].

The bitumen used in asphalt shingles is typically produced through a periodic oxidation process and is characterized by a higher oxidation degree compared to paving-grade bitumen. Therefore, it exhibits increased stiffness and reduced ductility [5]. An additional factor affecting bitumen properties is the aging process. Given that the average service life of asphalt shingles is approximately 25 years, the material is significantly more exposed to environmental factors, such as water and UV radiation, which accelerate the aging process [2,3]. Consequently, the bitumen recovered from TOAS is stiffer and more brittle compared to paving-grade bitumen, which limits its deformability and increases its susceptibility to cracking. These reasons may limit the maximum allowable amount of the TOAS and MWAS additives in asphalt mixtures [2,6]. One potential solution could be to differentiate material requirements based on the source of RAS [7].

Previous research on the use of RAS has largely focused on the properties of asphalt mixtures rather than on bitumen characteristics. TOAS has been used more extensively than MWAS [1]. The literature presents examples of varying results depending on the type or source of the RAS used. In a study of Casione et al. [8], it was shown that, depending on the testing region of experimental sections containing RAS, the application of TOAS led to either no change or an improvement in low-temperature cracking resistance. The same study also reported differences in the amount of transverse cracking depending on the RAS source. The mixtures containing MWAS exhibited more cracking compared to those with TOAS. Kanaan et al. [9] observed that the type of RAS used (regardless of whether it was TOAS or MWAS) caused changes in the rheological properties of the asphalt mastics tested compared to the mixture without the addition of RAS. Moreover, the increase in stiffness modulus and a decrease in phase angle were noted after RAS modification. In summary, RAS is a valuable material for use in asphalt mixtures; however, due to its production method and aging process, its proportion in the mixture must take into account the origin of the RAS. In asphalt mixtures, TOAS is used much more frequently than MWAS.

The RAS addition significantly stiffens the base bitumen—resulting in an increased stiffness modulus, higher viscosity, and an elevated upper Performance Grade (PG) limit. This contributes to the improved resistance of RAS-containing asphalt mixtures to permanent deformation [1,5,10,11,12,13,14]. Abbas et al. [14] observed that, within the tested range of up to 10% MWAS-derived bitumen, increasing the additive content led to a decrease in the J_nr_3.2 parameter and an increase in the G/sin(δ) value—both of which are indicators of enhanced resistance to permanent deformation. Similar research was conducted by D. J. Oldham et al. [15]. In that study as well, an increase in the G/sin(δ) parameter was observed, indicating the improved rutting resistance of TOAS-modified bitumen. Differences in rutting resistance, depending on the origin of the asphalt shingles (without distinction between TOAS and MWAS), were indicated by Alvergue et al. [16]. In their research, Sengoz et al. [17] found that adding 1% TOAS to an asphalt mixture significantly increased its rutting resistance. A similar effect, with a 5% TOAS addition, was confirmed by Aguirre et al. [2]. The impact of RAS-modified bitumen on deformation resistance is also supported by the results of the Linear Amplitude Sweep (LAS) test, carried out on bitumen modified with TOAS-derived bitumen [18]. In summary, the addition of RAS stiffens the base bitumen and improves its rutting resistance. Even small amounts of TOAS (1–5%) significantly enhance the durability of asphalt mixtures.

Regarding fatigue resistance, the influence of RAS additives on both the bitumen and mixtures containing these additives is varied. Some researchers report an improvement in fatigue resistance [9,19], while others observe its deterioration [20,21]. Testing conditions may significantly affect these outcomes. As was noted in study [9], fatigue life improves under controlled stress testing, whereas it decreases under controlled strain conditions.

At the same time, a reduction in low-temperature performance is observed. The mixtures containing RAS are more susceptible to low-temperature cracking [7,22,23], especially those incorporating the bitumen derived from TOAS due to the presence of more aged bitumen [12]. For this reason, some researchers recommend limiting the amount of RAS-derived bitumen to 30% of the mixture mass [7].

In Poland, the research on RAS reuse in asphalt mixtures is not widespread. Studies in this area have been conducted by Zieliński [4,10,24,25]. In these studies, MWAS shingle scraps at an amount of 4% by mixture mass were used as an additive in asphalt mixtures with 50/70 and 70/100 paving-grade bitumen. This research showed that RAS addition improves rutting resistance. No deterioration in low-temperature properties was observed, while fatigue resistance improved; however, the results varied depending on the test method. As can be seen, the experience with RAS in Poland is still limited and has focused mainly on the properties of asphalt mixtures. The present study concentrates on determining the effect of bitumen derived from TOAS and MWAS on paving-grade bitumen properties, which will make it possible to establish the optimal amount of this additive under Polish conditions. So far, research on the use of RAS has focused mainly on examining the properties of asphalt mixtures containing this additive. In most studies, only one type of RAS additive was evaluated: MWAS or TOAS. The study presents a comparison of the impact of bitumen derived from different sources of RAS on the properties of the base bitumen. The results of fatigue durability testing using the LAS method were also presented. Until now, this evaluation has been carried out for asphalt mixtures, and to a limited extent for bitumen.

## 2. Materials and Methods

### 2.1. Materials

The study utilized 50/70 paving-grade bitumen (PKN Orlen, Plock, Poland), which is widely used in asphalt mixtures subjected to traffic loads KR1–KR4. The modification of the paving-grade bitumen was carried out using recovered bitumen from two types of RAS. The first type—TOAS—originated from the demolition of a 25-year-old roof located in southeastern Poland, where the climate is temperate. Samples of roofing asphalt shingles were taken from several spots within the pile resulting from the demolition, and then mixed together to ensure uniformity. The second type included offcuts from new shingles, which are identical to the post-manufacturing waste asphalt shingles (MWAS). Additions of 15%, 30%, and 45% RAS-derived bitumen were applied relative to the total mass of the bitumen blend. This dosage range enables the assessment of its impact on the base bitumen, reflecting the typical levels applied in asphalt mixtures. The modification procedure of base bitumen is described in Section 2.2.1. The basic properties of the 50/70 paving-grade bitumen (I) and the recovered bitumen from both TOAS (II) and MWAS (III) are summarized in Table 1.

### 2.2. Methods

#### 2.2.1. Bitumen Modification

**Figure 1:**
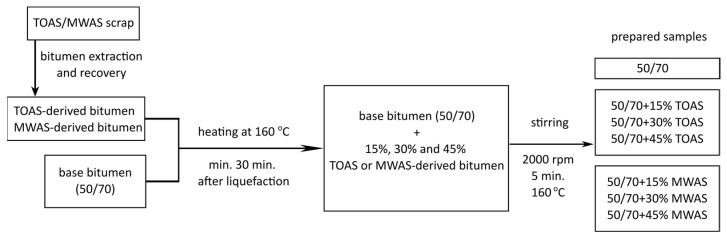
Flow chart of modified bitumen preparation.

The bitumen recovered from RAS was extracted using an automatic extractor (Infratest, Brackenheim, Germany), in accordance with EN 12697-1 [29], and then the solvent—tetrachloroethylene (Chempur, Piekary Slaskie, Poland)—was removed using a rotary evaporator (Heidolph, Schwabach, Germany), in accordance with EN 12697-3 [30]. Approximately 1 L of bitumen solution was gradually transferred into a rotating flask (75 rpm) and immersed in an oil bath at 110 °C. The pressure in the evaporator system was maintained at 40 kPa. Once the entire solution had been transferred, the oil bath temperature was increased to 160 °C, and the pressure was reduced to 2 kPa. These conditions were held until the cessation of bubble formation, indicating that solvent removal was complete. If bubbling continued after 10 min, the bath temperature was increased further to 180 °C and maintained until bubbling ceased entirely. The total recovery time for each batch was monitored and kept below 2 h. After sufficient bitumen was recovered, the samples from multiple distillation runs were combined in a large container and homogenized through thorough mixing. This step was performed to achieve uniformity of the recovered bitumen for use in further testing. The bitumen recovered from the RAS, together with base bitumen, were heated in an oven to a temperature of 160 °C (until both types of bitumen became liquefied and had been kept at that state for 30 min), and then mixed in appropriate proportions (15%, 30%, 45%), using a homogenizer for 5 min with a mixing speed of 2000 rpm [5]. The TOAS and MWAS percentages were selected based on previous studies conducted for asphalt mixtures [16]. Throughout the modification process, the sample was maintained at a constant temperature using a heating jacket. After mixing, the samples were poured into containers or molds in accordance with the relevant standard. The process of modifying the base bitumen with TOAS/MWAS-derived bitumen is shown in Figure 1.

#### 2.2.2. Test of Physical Properties

The penetration test was carried out in accordance with the EN 1426 [26] standard at a temperature of 25 °C in order to assess the hardness of the received modified bitumen. The test consists of measuring the depth of penetration of a needle into the bitumen sample under a load of 100 g over a period of 5 s.

The softening point test was performed in accordance with the EN 1427 [27] standard, using water. If the softening point was found to be higher than 80 °C, the test was repeated using glycerin.

The Penetration Index (*PI*) is the simplest measure of the thermal sensitivity of bitumen and was calculated based on the penetration at 25 °C and the softening point temperature, assuming that the penetration at the softening point is 800 × 0.1 mm, in accordance with Equation (1) specified in the EN 12591 [31] standard. A lower *PI* value means that bitumen changes its consistency more rapidly with temperature changes [32].

(1) $$PI=\frac{20T_{R\&B}+500\log\left(PEN\right)-1952}{T_{R\&B}+50\log\left(PEN\right)-120}$$

where:

*PI*—Penetration Index (−),
*T_R_*_&*B*_—softening point (°C),
*PEN*—penetration at 25 °C (0.1 mm).

#### 2.2.3. Viscosity Test

The viscosity test was performed using a Brookfield viscometer (AMETEK Brookfield, Middleborough, MA, USA) in accordance with the ASTM D4402 [33] standard at three temperatures: 90 °C, 135 °C, and 160 °C. Before measurement, the investigated bitumen was thermostated at the test temperature for at least 30 min to stabilize the measurement temperature.

#### 2.2.4. MSCR Test

The MSCR test was performed according to AASHTO T 350-19 [34], using a DSR rheometer (Anton Paar, Graz, Austria) with a plate-plate system of the following geometry: 25 mm diameter and 1 mm sample thickness. The MSCR test involves repeatedly loading the bitumen sample for 1 s followed by a 9 s recovery period. Each cycle was repeated 10 times at two stress levels: 0.1 kPa and 3.2 kPa [34]. On the basis of this, the J_nr_3.2 value was calculated, which defines the resistance of the tested bitumen to permanent deformation. The lower its value, the greater the potential rutting resistance of the asphalt mixture made with the bitumen. The test was conducted at a temperature of 64 °C, which—under Polish climatic conditions—shows the best correlation with rutting test results [35]. An additional parameter is the percent recovery R (%), which is an indicator of bitumen elasticity. The higher the R value, the more elastic the bitumen [36].

#### 2.2.5. LAS Test

The LAS (Linear Amplitude Sweep) test was carried out according to AASHTO T 391-20 [37], using a DSR rheometer (Anton Paar, Graz, Austria) with a plate-plate system of 8 mm diameter and a 2 mm sample thickness at a temperature of 15 °C, adopted as the equivalent temperature of asphalt layers in semi-rigid pavements. This test assesses the bitumen fatigue life and is based on the VECD (Viscoelastic Continuum Damage) model. It consists of two stages: first, a frequency sweep test is performed in the range of 0.2 to 30 Hz at a strain level of 0.1%, followed by a strain sweep test at a constant frequency of 10 Hz [38,39]. On the basis of these results, parameters *A, B*, and the fatigue life $N_{f}$ are calculated, which can be described by Equation (2):

(2) $$N_{f}=A{\gamma_{max}}^{-B}$$

where:

*N_f_*—fatigue life,
*A*, *B*—fatigue life equation parameters,
$\gamma_{max}$—maximum expected bitumen strain (%).

#### 2.2.6. FTIR Analysis

FTIR spectra of both the base and TOAS/MWAS-modified bitumen were obtained using the ATR (Attenuated Total Reflectance) technique with a Nicolet 380 spectrometer. Each spectrum was recorded at a resolution of 4 with a scan number of 32. During the measurement, the bitumen was taken analytically and placed on an ATR crystal. Spectra were recorded at room temperature without additional preparation. On the basis of the recorded spectra, quantitative indices were determined to describe the content of functional groups: –OH (I_–OH_), C=O (I_C=O_), S=O (I_S=O_), linear hydrocarbons (I_–CH3/–CH2−_), and cross-linked hydrocarbons (I_–CH3_).

(3) $$I_{-OH}=\frac{A_{3400}}{\sum A}$$

(4) $$I_{C=O}=\frac{A_{1700}}{\sum A}$$

(5) $$I_{S=O}=\frac{A_{1030}}{\sum A}$$

(6) $$I_{-CH3/-CH2-}=\frac{A_{1460}}{\sum A}$$

(7) $$I_{-CH3}=\frac{A_{1380}}{\sum A}$$

where,

$I_{-OH}$—hydroxyl index

$A_{3400}$—area of peak at 3400 cm^−1^ wavelength

$I_{C=O}$—carbonyl index

$A_{1700\;}$—area of peak at 1700 cm^−1^ wavelength

$I_{S=O}$—sulfoxide index

$A_{3400\;}$—area of peak at 1030 cm^−1^ wavelength

$I_{-CH3/-CH2-}$—linear hydrocarbon index

$A_{1460}$—area of peak at 1460 cm^−1^ wavelength

$I_{-CH3}$—branched hydrocarbon index

$A_{1380}$—area of peak at 1380 cm^−1^ wavelength

∑A-sum of peak areas at FTIR spectra

## 3. Results and Discussion

### 3.1. Neat Bitumen and TOAS- and MWAS—Modified Bitumen Physical Properties

**Figure 2:**
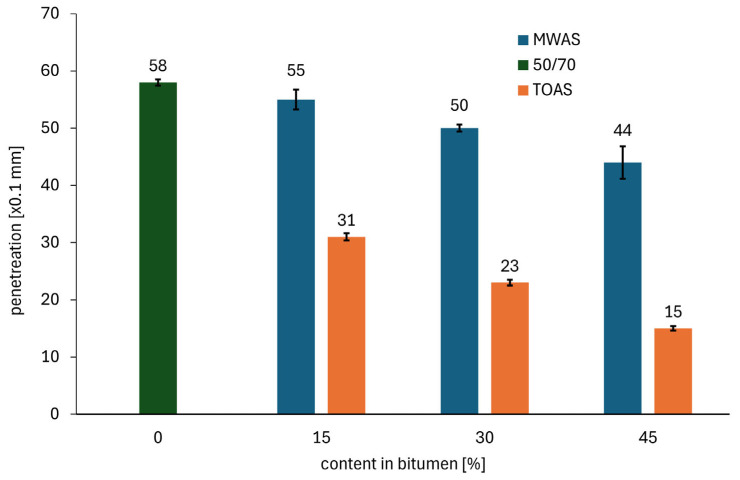
Penetration of the bitumen blend.

**Figure 3:**
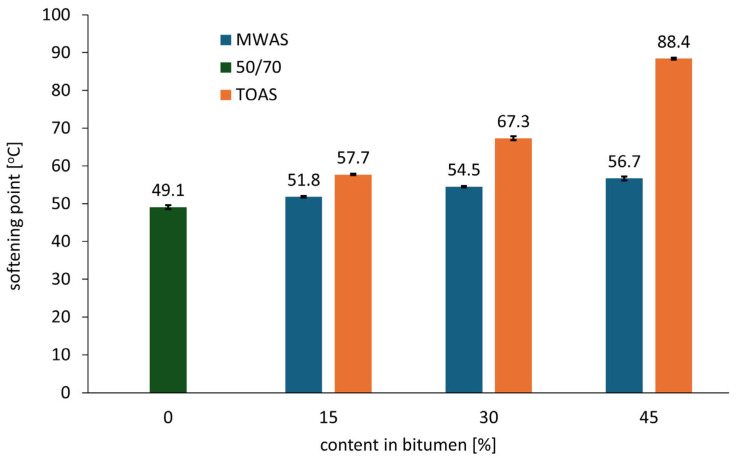
Softening point of the bitumen blend.

Changes in the penetration and softening point of 50/70 paving-grade bitumen depending on the TOAS- and MWAS-derived bitumen amount are presented in Figure 2 and Figure 3, respectively.

On the basis of the analysis of the results presented in Figure 2 and Table 1, the origin of the RAS has a significant impact on the properties of the bitumen blend. In particular, the use of TOAS-derived bitumen leads to a considerably greater reduction in penetration compared to MWAS-derived bitumen. As can be seen in Figure 2, 15% addition of bitumen recovered from TOAS reduces the penetration of the base bitumen by 46% (from 58 × 0.1 mm to 31 × 0.1 mm), whereas application of bitumen from MWAS causes only a 5% decrease (from 58 × 0.1 mm to 55 × 0.1 mm). The analysis of the relationship shown in Figure 2 also highlights the limitations of using both types of RAS. The addition of recovered bitumen from MWAS does not significantly reduce the penetration, even at a 45% addition level (resulting in a change in bitumen grade from the original 50/70 to 35/50—thus one grade harder according to [31]). However, a relatively small amount of recovered bitumen from TOAS results in a significant hardening of the base bitumen (changing from the original 50/70 grade to just above the upper limit for the 20/30 grade—thus, two grades harder according to [31]). Further increases in the additive cause the penetration value to decrease to 15 × 0.1 mm, which may lead to increased susceptibility of the bitumen to low-temperature cracking.

Similarly to the penetration results, the softening point also indicates hardening of the tested bitumen. An increase in the proportion of RAS-derived bitumen results in a corresponding increase in the softening point. Similar to penetration, the change in softening point depends on the type of RAS used. In the case of the TOAS-derived bitumen, a much greater increase is observed compared to the MWAS-derived bitumen. Its application at the maximum addition amount of 45% *w*/*w* MWAS-derived bitumen results in a softening point increase of 7.6 °C (an increase of 15.5%), while the same amount of TOAS-derived bitumen causes an increase of 39.3 °C (an increase of 80%). This is due to the properties of the recovered bitumen. As is presented in Table 1, the TOAS-derived bitumen has a softening point approximately 50% higher than that of the MWAS-derived bitumen.

**Figure 4:**
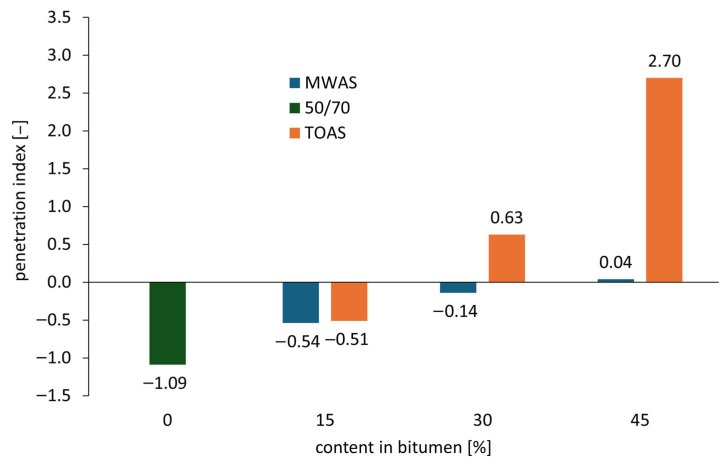
Penetration index of the bitumen blend.

Figure 4 presents the Penetration Index (*PI*) values for the tested bitumen, determined using Equation (1).

Figure 4 clearly indicates a significant impact of the applied bitumen on the Penetration Index (*PI*) value of the modified 50/70 paving-grade bitumen. The initial *PI* of the base 50/70 paving-grade bitumen value of −1.09 indicates high sensitivity to temperature changes. As the content of RAS-derived bitumen increases, an improvement in this parameter is observed—the *PI* value increases. The greatest change is observed for the bitumen recovered from TOAS. For 50/70 paving-grade bitumen with 45% additive, the *PI* reaches 2.70, while for the same content of bitumen from MWAS, the Penetration Index remains close to 0. On the basis of this, conclusions can be drawn regarding the colloidal structure of the tested bitumen. For additions up to 30%, the tested bitumen types are expected to exhibit a SOL-GEL type structure—retaining viscoelastic properties over a wide temperature range, with resistance to deformation and aging. The base bitumen mixed with MWAS-derived bitumen at an amount of 45% still retains these properties, while the bitumen containing TOAS-derived bitumen shifts toward a GEL-type structure. Although such bitumen types maintain high resistance to temperature changes, they may exhibit brittle behavior at low temperatures. This finding is consistent with the results obtained for penetration and softening point; these are hard bitumen types with high viscosity. Thus, it can be assumed that the addition of more than 30% TOAS-derived bitumen should not be used due to its negative impact on the base bitumen.

### 3.2. Viscosity Test Results

**Figure 5:**
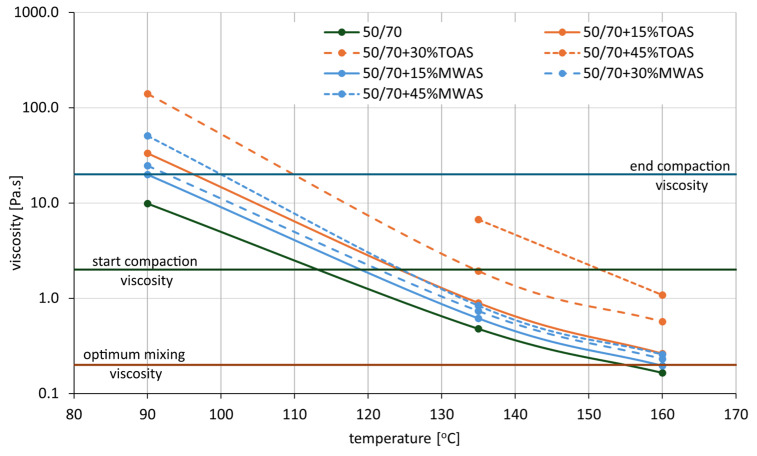
Viscosity of the bitumen blend.

The results of the viscosity tests for paving-grade bitumen with the addition of bitumen recovered from RAS are presented in Table 2 and Figure 5. The tests were conducted at three temperatures: 90 °C, 135 °C, and 160 °C. For the sample containing 45% TOAS-derived bitumen, the test could not be performed due to equipment limitations. In this case, viscosity was beyond the measurement range of the device.

Analysis of the results presented in Figure 5 reveals a significant impact of the addition of bitumen recovered from RAS on the viscosity values of 50/70 paving-grade bitumen. The influence of the recovered bitumen is particularly evident at lower test temperatures and depends on the type of RAS used. In the case of the bitumen from MWAS, the increase in viscosity is significantly smaller than in the case of the bitumen from TOAS.

At 160 °C, the addition of MWAS-derived bitumen caused only a slight increase in viscosity. This increase ranged from 20% to 60% for 15% and 45% MWAS-derived bitumen additions, respectively (from 0.165 Pa·s to 0.196 Pa·s for 15%, and to 0.257 Pa·s for 45%). In contrast, the bitumen recovered from TOAS showed a much greater increase in viscosity. For the TOAS-derived bitumen addition at an amount of 15%, the increase was approximately 60% (up to 0.263 Pa·s), while for a 45% addition, the viscosity rose by over 600% (up to 1.080 Pa·s). A similar trend was observed at the other two temperatures. Again, the viscosity increase was much more significant when the TOAS-derived bitumen was used compared to the MWAS-derived bitumen. At 135 °C, the viscosity increase for the MWAS-derived bitumen additions was similar to that observed at 160 °C, while for the TOAS-derived bitumen, the increase was approximately 1400% (from 0.478 Pa·s for base bitumen to 6.700 Pa·s).

Such substantial increases in viscosity pose challenges during the production and laying of asphalt mixtures with this bitumen. The optimal bitumen viscosity for mixing is considered to be around 0.2 Pa·s, and during compaction, it should range between 2 and 20 Pa·s [40]. For the base 50/70 paving-grade bitumen, the optimal mixing temperature is approximately 155 °C. With a 15% addition of the MWAS-derived bitumen, the mixing temperature increases to around 160 °C, while for 30% and 45% MWAS-derived bitumen additions, the optimal mixing temperature is expected to be around 170 °C. This indicates an increase in the optimal mixing temperature by 5 to 15 °C. For the TOAS-derived bitumen additions, these temperatures are significantly higher. A 15% addition of the TOAS-derived bitumen requires a mixing temperature of around 170 °C, and even higher temperatures are necessary for greater additions. This results in increased costs due to the need to heat both the mineral aggregate and the bitumen to higher temperatures than those used for standard mixtures. It also highlights the need to either use viscosity-reducing agents (like zeolites or F-T waxes) when higher amounts of RAS are added to the asphalt mixture or to limit the amount of such additives. Of course, the appropriate solution depends on the origin of the recovered RAS used. Another aspect associated with the increased viscosity of the bitumen (and the resulting higher mixing temperature) is the elevated emission of polycyclic aromatic hydrocarbons (PAHs). At standard mixing temperatures, the emission of PAHs containing two- or three-rings is observed. An increase in temperature leads to the release of compounds with four- to six-rings. This effect is further intensified by the high degree of oxidation of the bitumen derived from RAS [41]. Additionally, the compaction temperature range of the mixture shifts when RAS is added. For base bitumen, compaction should begin once the mixture reaches approximately 112 °C, and end well below 90 °C. With a 15% MWAS-derived bitumen addition, the effective compaction range shifts to around 120 °C at the start and 90 °C at the end. Increasing the MWAS-derived bitumen content to 45% shifts this range to 100–125 °C. A similar range is observed for 15% TOAS-derived bitumen addition (96–125 °C), while for 30% TOAS-derived bitumen, the compaction range extends to 110–135 °C.

### 3.3. MSCR Test Results

**Figure 6:**
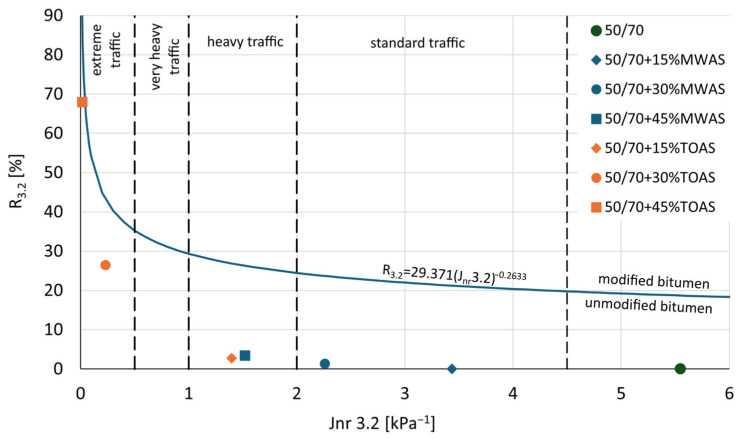
MSCR test results: Recovery (R) as a function of non-recoverable compliance (J_nr_) at a 3.2 kPa load and 64 °C.

To determine the properties of the bitumen at high temperatures, the MSCR test was used. This test makes it possible to assess the contribution of the bitumen to rutting resistance. The graph in Figure 6 presents the obtained values of the J_nr_3.2 and R parameters at a stress level of 3.2 kPa.

**Figure 7:**
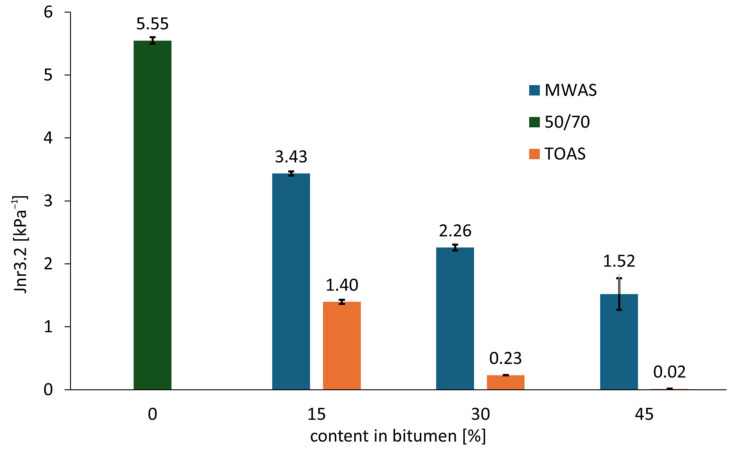
J_nr_3.2 compliance as a function of recovered RAS bitumen content at 3.2 kPa load.

**Figure 8:**
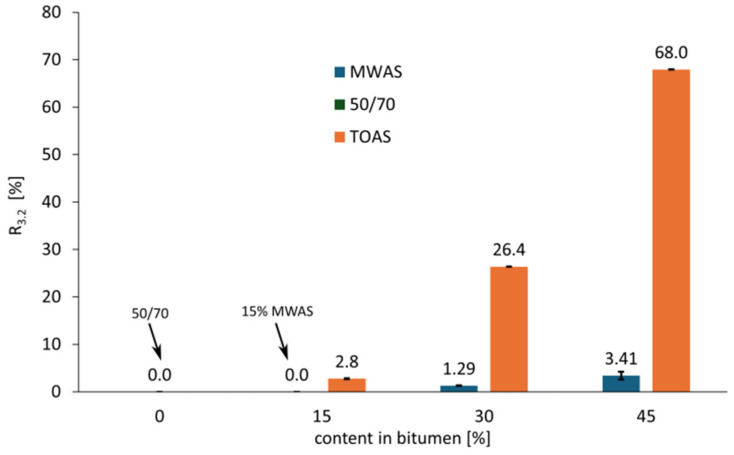
Recovery R_3.2_ compliance as a function of recovered RAS bitumen content at 3.2 kPa load.

On the basis of the analysis of the graph showed on Figure 6, it can be concluded that all tested bitumen types are unmodified (i.e., they do not meet the requirements for modified bitumen with respect to the R_3.2_–J_nr3.2_ relationship). This is evidenced by their position below the reference line, which was adopted based on literature sources [42,43] and separates modified from unmodified bitumen. The 50/70 paving-grade bitumen shows high susceptibility to rutting, as it falls outside the acceptable range for standard traffic (<3 million ESAL according to [44]). As is shown in Figure 7 and Figure 8, for the reference and the MWAS-modified bitumen in a ratio of 15%, the R3.2 value was determined to be 0%, which is possible and has also been pointed out by other researchers [45]. The use of the recovered bitumen from MWAS improves rutting resistance. As is seen in Figure 7 and Figure 8, the MWAS-derived bitumen additions in amount of 15% and 30% cause a decrease in the J_nr_3.2 parameter and simultaneously a slight increase in recovery R_3.2_. These bitumen types meet the requirements for standard traffic conditions according to [44]. Increasing the MWAS-derived bitumen content to 45% results in further improvements in rutting resistance, along with a noticeable increase in the R_3.2_ value. This bitumen meets the criteria for heavy traffic (3–10 million ESAL according to [44]). The addition of the bitumen recovered from TOAS significantly enhances the base bitumen resistance to permanent deformation. A 15% addition achieves similar performance to a 45% addition of the MWAS-derived bitumen. Increasing the TOAS-derived bitumen content to 30% and 45% leads to a substantial reduction in the J_nr_3.2 parameter and an increase in recovery R_3.2_. Bitumen with this level of the TOAS-derived bitumen addition meets the requirements for extreme traffic conditions (over 30 million ESAL according to [44]). To better illustrate the influence of RAS recovered bitumen on base bitumen properties, Figure 7 shows the relationship between the amount of recovered bitumen and the J_nr_3.2 parameter, while Figure 8 presents the relationship between the additive content and the R_3.2_ parameter.

On the basis of the analysis of the graph shown in Figure 7, a significant influence of the addition of bitumen recovered from RAS—as well as its source—can be observed. The origin of the RAS has the greatest impact. For bitumen recovered from TOAS, the reduction in the J_nr_3.2 parameter reaches 74% for a 15% addition, 96% for a 30% addition, and nearly 99% for a 45% addition. Due to the very low J_nr_3.2 value for the 45% TOAS-derived bitumen addition, which falls outside the precision range of the MSCR method, the result should be interpreted with great caution. In comparison, the bitumen recovered from MWAS results in smaller reductions of 38%, 59%, and 72%, respectively, for 15%, 30%, and 45% additions. The use of recovered RAS bitumen also increases the recovery R_3.2_ of the tested bitumen, as illustrated in Figure 8. For the base 50/70 paving-grade bitumen, the R_3.2_ value is zero. The addition of MWAS-derived bitumen does not significantly improve this parameter; noticeable effects appear only at a 30% addition, with a modest value of around 1.3%. A 45% addition results in a more than two-fold increase, reaching 3.41%. In contrast, the addition of the TOAS-derived bitumen has a greater impact. A 15% addition increases recovery to 2.80%, which is still relatively low. However, increasing the amount to 30% leads to a substantial rise in the R_3.2_ parameter—nearly a ten-fold increase to 26.4%. A further increase to 45% results in a 2.5-fold increase in recovery, reaching 68%. Notably, the difference in R_3.2_ values between the 30% and 45% additions is approximately twenty-fold. An increase in the R_3.2_ parameter, combined with a decrease in J_nr_3.2, indicates greater elasticity alongside increased stiffness of the bitumen modified with the TOAS- or MWAS-derived bitumen compared to the base bitumen. This should positively affect the permanent deformation resistance of asphalt mixtures incorporating these bitumen types.

### 3.4. LAS Test Results

**Figure 9:**
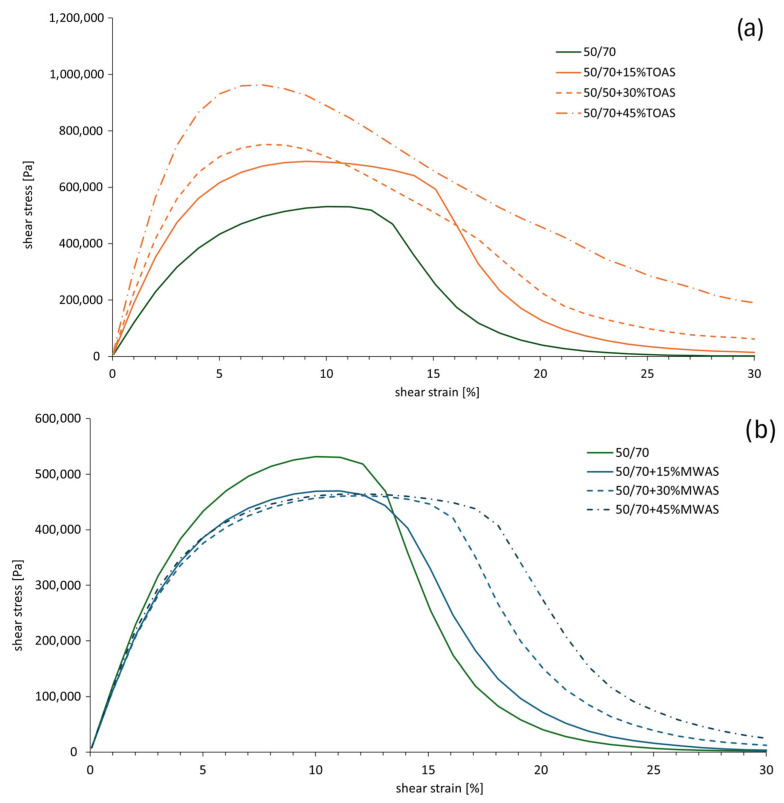
Stress–strain curve from the LAS test: (**a**) bitumen with the TOAS-derived bitumen additive, (**b**) bitumen with the MWAS-derived bitumen additive.

**Figure 10:**
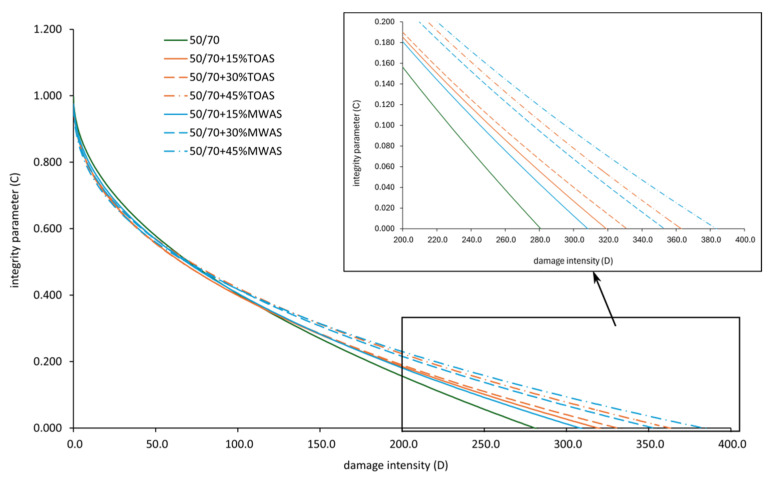
Material integrity curve as a function of damage intensity from the LAS test.

The LAS (Linear Amplitude Sweep) test was conducted to determine the resistance of the tested bitumen to fatigue damage. The failure criterion is defined as a 35% reduction in the G*sind parameter [46]. Figure 9 presents the averaged stress–strain experimental curves, while Figure 10 shows the damage curve.

The analysis of the curves indicates a different influence of RAS from various sources on the obtained test results. It can be observed that with an increase in the RAS-derived bitumen content, the value of parameter C increases. In general, for the same amounts of the RAS-derived bitumen additive, the bitumen with the MWAS-derived bitumen achieves higher values of parameter C than the 50/70 paving-grade bitumen with the TOAS-derived bitumen. The only exception is for the 15% additive, where the bitumen with the TOAS-derived bitumen attains a higher value of parameter C compared to the 50/70 paving-grade bitumen with the MWAS-derived bitumen. While analyzing the graph shown in Figure 10, it can be seen that with an increase in the RAS-derived bitumen additive, the material integrity parameter decreases more slowly, which means a slower reduction of the dynamic shear modulus. For the same value of parameter C, an increase in the corresponding parameter D leads to an increase in the number of cycles the bitumen can endure until failure, thereby improving its fatigue life [47]. Hence, it can be concluded that the addition of the RAS-derived bitumen should enhance the fatigue durability of the tested bitumen blends. Figure 9 shows the different behaviors of the tested bitumen. For the base 50/70 paving-grade bitumen, the maximum stress value is reached at about 11% strain, after which, there is a rather rapid decline in shear stress. In the case of the bitumen with the MWAS-derived bitumen additive, slightly lower maximum stresses are observed, approximately 86 to 88% of the stress of the pure bitumen. The strain level at which these stresses are reached is similar to that of the base bitumen, about 11–12%. However, unlike the base bitumen, the decline in stress with increasing strain is less steep. As the amount of the MWAS-derived bitumen additive increases, this decline becomes more gradual, indicating that the bitumen can still carry some load after damage. In the case of the bitumen with TOAS-derived bitumen additive, a significant increase in shear stress values is observed. The increase ranges from 30% to 80% for 15% and 45% additives, respectively. However, the strain at which the peak stress occurs decreases. For 15% TOAS-derived bitumen additive, the peak stress is reached at about 9% strain, and for 30% and 45% additives, at about 7% strain. After reaching the peak stress, the shear stress declines relatively quickly. At around 17% strain, the stress values for 15% and 30% TOAS-derived bitumen additives are similar to those for the MWAS-derived bitumen. The stress level for 45% TOAS-derived bitumen additive decreases relatively slowly; at a maximum strain of 30%, it remains four-times higher than the stress of the bitumen with 45% MWAS-derived bitumen additive.

The fatigue life function is characterized by parameters *A* and *B*, the values of which are presented in Table 3.

**Figure 11:**
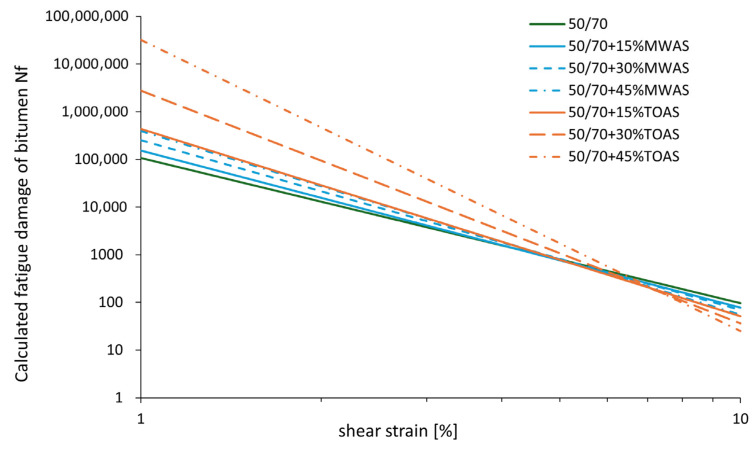
Number of cycles to failure as a function of strain from the LAS test.

The value of parameter *A* corresponds to the initial fatigue life at a strain level of 1%. Therefore, an increase in parameter *A* results in a higher estimated fatigue life of the bitumen. Parameter *B*, on the other hand, depends on the slope “m” of the dynamic shear modulus-frequency curve obtained from the “frequency sweep” test. A decrease in the absolute value of parameter *B* indicates an increase in the fatigue life of the tested bitumen blends. Table 3 shows that the addition RAS-derived bitumen significantly increases the fatigue life of the base bitumen. This is evident from the increases in both parameter *A* and parameter *B*. This is also reflected in the values of the number of cycles to failure, *N_f_*, at strains of 2.5% and 5%, which are also presented in Table 3. For the bitumen reclaimed from TOAS, at 2.5% strain, an increase in fatigue life of 81% is observed for 15% additive, 383% for 30% additive, and 1728% for 45% additive. In the case of the bitumen from MWAS, the increase in fatigue life is much smaller—only 14%, 47%, and 75% for 15%, 30%, and 45% additives, respectively. At 5% strain, the addition of the TOAS-derived bitumen causes a slight decrease in fatigue life by 2% for the 15% additive, while for 30% and 45% additives, an increase of 36% and 120% is observed compared to the base bitumen. For the bitumen from MWAS, the difference between the base bitumen and the one with the RAS-derived bitumen additive is small, about 3–4%. The fatigue life dependence described by Equation (2)—fatigue life as a function of strain—is shown in Figure 11. Here, the relationships described by the parameters in Table 3 are confirmed. The highest fatigue life values were obtained for bitumen with the addition of bitumen from the TOAS-derived bitumen, while lower values were obtained with bitumen containing the bitumen from MWAS. In every case, an increase in fatigue life is observed.

It is worth noting that despite increasing fatigue life, the addition of the TOAS-derived bitumen shows an unfavorable trend with respect to applied strain. With increasing strain, the fatigue life decreases very rapidly. Changing the strain level from 2.5% to 5.0% for this bitumen results in a 98% reduction in fatigue life, while for the analogous 45% MWAS-derived bitumen additive, the decrease is 93%. For the base 50/70 paving-grade bitumen, the change in strain level from 2.5% to 5.0% results in an 88% decrease in fatigue life. The stiffening of the base bitumen caused by the addition of the hard bitumen derived from TOAS or MWAS results in improved fatigue life at low strain levels. However, as the strain increases, fatigue life decreases—this effect is particularly noticeable for the base bitumen containing the TOAS-derived bitumen. This is related to the significant degree of aging of this bitumen—the bitumen becomes more brittle, and its fatigue life becomes more susceptible to strain level. In the case of the MWAS-derived bitumen, the bitumen is not as aged as the TOAS-derived bitumen, which is why the change in the fatigue life of the base bitumen with this additive is not as significant as in the case of the TOAS-derived bitumen.

### 3.5. FTIR Analysis Results

**Figure 12:**
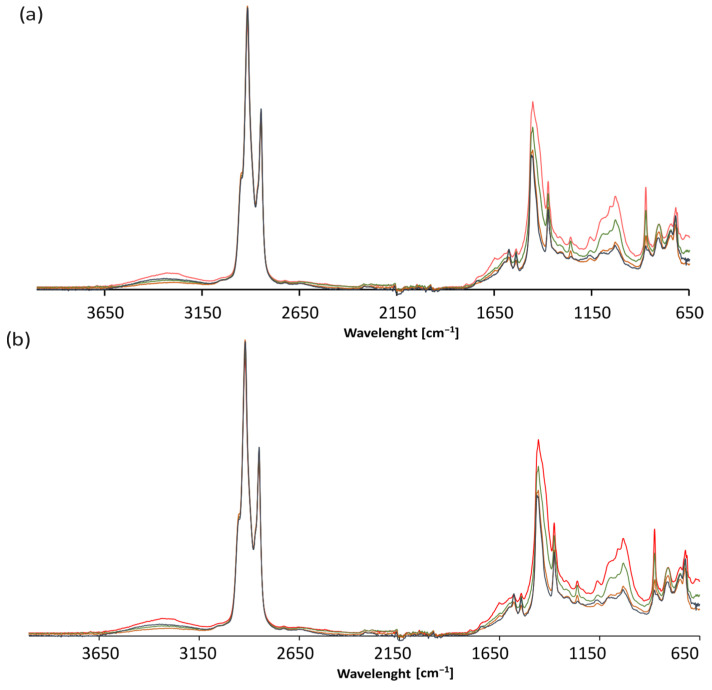
FTIR spectra of neat bitumen (blue line) and the bitumen modified with 15% (orange line), 30% (green line), and 45% (red line) addition of TOAS (**a**) and MWAS (**b**).

**Figure 13:**
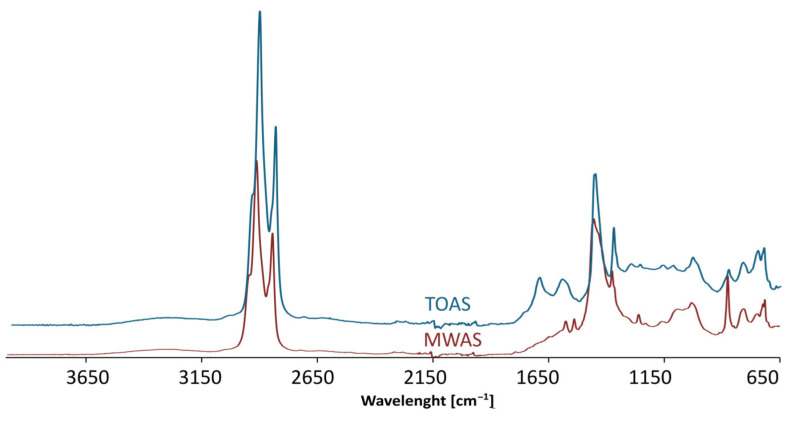
FTIR spectra of MWAS and TOAS implemented for bitumen modification.

**Figure 14:**
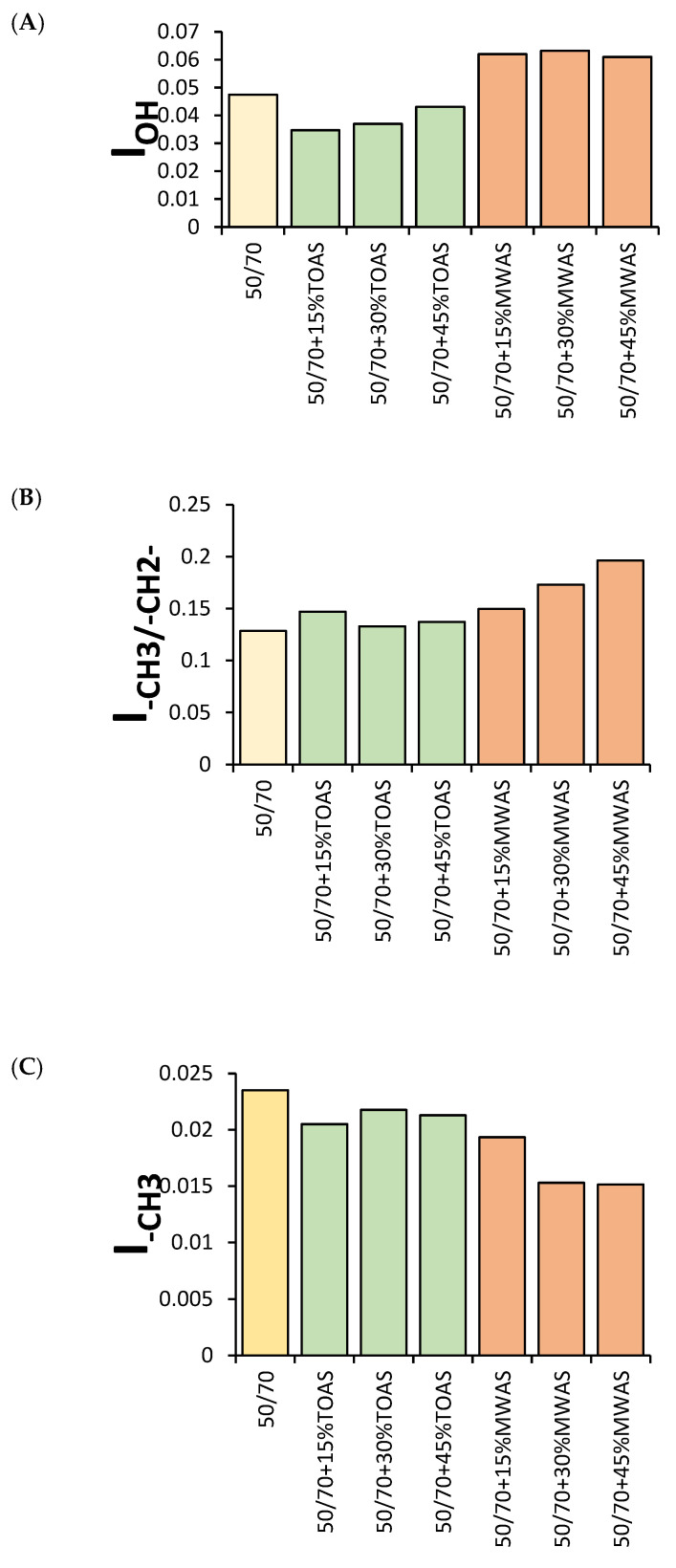

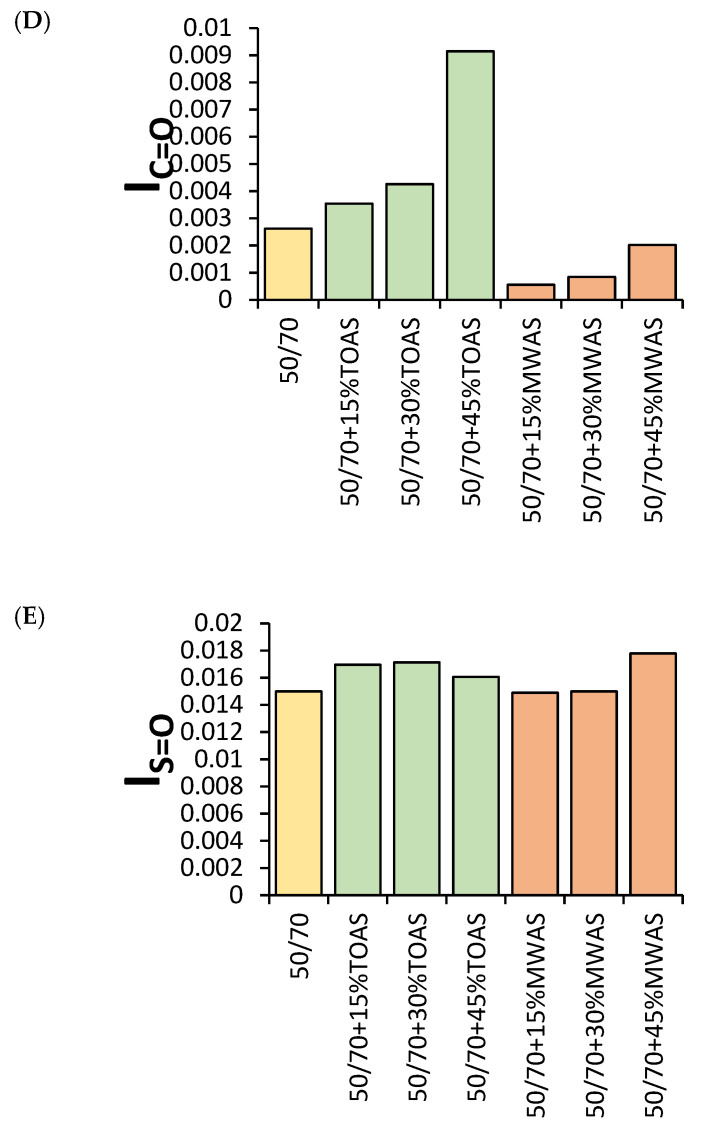
I_OH_ (**A**), I_–CH3/–CH2−_ (**B**), I_–CH3_ (**C**), I_C=O_ (**D**), and I_S=O_ (**E**) of the 50/70 bitumen before and after TOAS/MWAS modification.

A structural analysis of the base bitumen and the bitumen modified with the MWAS- and TOAS-derived bitumen was then carried out. As can be seen in Figure 12a, the application of the TOAS-derived bitumen affects the chemical structure of the modified bitumen. The spectra recorded for the base 50/70 paving-grade bitumen and both the TOAS (Figure 13) and TOAS-modified bitumen (Figure 12a) at 15%, 30%, and 45% indicate different contents of –OH groups present in the structure of the bitumen tested. This is evident from the band located at 3400 cm^−1^, attributed to the O–H bond stretching vibrations [48]. However, this effect was not observed for the MWAS-modified bitumen (Figure 12b), despite the presence of these groups on the MWAS spectrum (Figure 13). The study carried out clearly indicates that –OH groups were present in the TOAS structure, as a systematic increase in the band originating from their vibrations was observed for increasing TOAS contents. This is also indicated by the increase in I_OH_ shown in Figure 14a. Furthermore, the FTIR analysis carried out showed that modification of 50/70 paving-grade bitumen with the TOAS-derived bitumen also affects the ratio of branched and linear hydrocarbons. This is evident in Figure 12a by a systematic increase in the band located at 1460 cm^−1^, attributed to the stretching vibrations of the –CH_3_, –CH_2−_ groups of linear hydrocarbons, with a simultaneous decrease in the intensity of the bands located at 1360 cm^−1^, attributed to the stretching vibrations of the –CH_3_ groups of branched hydrocarbons [49]. This is demonstrated by an increase in the I_CH3/–CH2−_ index (Figure 14b) and a decrease in the I_–CH3_ index (Figure 14c). FTIR and IC=O index (Figure 14d) analyses also showed that modification of the bitumen with the TOAS-derived bitumen increases the number of carbonyl groups in the form of esters and carboxylic acids. This is evidenced by a systematic increase in the peaks located at 1260 cm^−1^ and 1060 cm^−1^, attributed to C–O bond stretching vibrations occurring in esters and carboxylic acids, respectively. However, as can be seen in Figure 12b and Figure 14, modification of the MWAS-derived bitumen also causes an increase in the number of C=O groups in the form of carboxylic acids, which is evident from the increase in the peak located at a wavelength of 1700 cm^−1^ (Figure 12b). At the same time, the number of S=O groups does not change. For all the FTIR spectra recorded and determined from them vales I_S=O_ (Figure 14e), the peak located at 1030 cm^−1^ was comparable [50].

## 4. Conclusions

The circular economy approach assumes the reuse of waste materials, minimizing the generation of unused fractions. The use of RAS aligns with this trend. Currently, there are two primary methods of RAS disposal: the production of bituminous conglomerate or incineration. Due to the high bitumen content in RAS, which can be reused, incineration represents a waste of valuable resources. Utilizing RAS can reduce the consumption of new materials in road pavement construction, including both bitumen and fine aggregate. In the context of rising construction material prices, this may generate significant cost savings for investors. Additionally, the use of RAS helps reduce air pollutant emissions, contributing to climate protection and lowering waste management costs.

On the basis of the conducted research, the following conclusions can be drawn:

- The addition of RAS stiffens the base bitumen (reduced penetration and increased softening point). This effect depends on the type of additive and is more pronounced for TOAS than for MWAS.
- The addition of RAS improves bitumen resistance to permanent deformation. This is evident from the MSCR test, where the inclusion of RAS reduces the J_nr_3.2 parameter while increasing the recovery R_3.2_.
- The use of RAS enhances fatigue durability. In the LAS test, an increase in the number of cycles to failure at specific strain levels is observed. However, the use of RAS also increases the susceptibility of bitumen to changes in strain levels.
- On the basis of the conducted research, it can be concluded that the addition of TOAS should not exceed 30%, while MWAS can be used at up to 45% without significant deterioration of the base bitumen parameters. These values should also take into account the low-temperature properties of the tested bitumen, which should be the subject of further research.

## 5. Future Perspectives

The presented studies do not fully describe the analysis of the properties of the bitumen with the addition of the RAS-derived bitumen. However, they constitute a starting point for further work, which will include the determination of low-temperature properties (such as stiffness modulus and flexural creep stiffness) that, due to the high stiffness of the tested bitumen types as well as their short- and long-term aging processes, may significantly deteriorate. The use of rejuvenators can improve resistance to low-temperature cracking, and the selection of an appropriate additive as well as its dosage will be part of the planned research. At the same time, the studies will be extended to other types of bitumen: multigrade and polymer-modified bitumen. The application of quantitative analysis using thin-layer chromatography with flame ionization detection (TLC-FID), as well as the determination of the molecular size distribution of bitumen components using gel permeation chromatography (GPC), will allow for a better understanding of the modification processes of bitumen with the binder recovered from RAS.

## Figures and Tables

**Table 1:** Neat bitumen and TOAS- and MWAS-derived bitumen properties.

Property	Test Method	Results		
		I (50/70)	II (TOAS)	III (MWAS)
Penetration at 25 °C (0.1 mm)	PN-EN 1426 [26]	58	12	29
Softening point (°C)	PN-EN 1427 [27]	49.1	122.6	82.5
Fraass breaking point (°C)	PN-EN 12593 [28]	−15	NT *	NT *

* NT—not tested.

**Table 2:** Viscosity of the bitumen blend.

Bitumen	Viscosity at Test Temperature (Pa∙s)		
	90 (°C)	135 (°C)	160 (°C)
50/70	9.9	0.478	0.165
50/70+15%TOAS	33.1	0.893	0.263
50/70+30%TOAS	139.4	1.925	0.568
50/70+45%TOAS	NT *	6.700	1.080
50/70+15%MWAS	19.9	0.615	0.196
50/70+30%MWAS	24.6	0.735	0.231
50/70+45%MWAS	50.7	0.835	0.257

* NT—not tested.

**Table 3:** LAS test parameters.

Bitumen	Parameters					
	α	*A*	*B*	*N_f_* 2.5%	*N_f_* 5.0%	*N_f_* 10.0%
50/70	1.525	107,671	−3.049	6587	796	96
50/70+15%TOAS	1.965	437,616	−3.930	11,945	784	51
50/70+30%TOAS	2.443	2,779,120	−4.884	31,829	1083	36
50/70+45%TOAS	3.055	32,433,456	−6.110	120,433	1747	25
50/70+15%MWAS	1.649	153,868	−3.298	7495	762	78
50/70+30%MWAS	1.781	253,145	−3.562	9648	820	69
50/70+45%MWAS	1.928	395,673	−3.857	11,552	798	55

## Data Availability

The original contributions presented in this study are included in the article material. Further inquiries can be directed to the corresponding authors.

## Abbreviations

The following abbreviations are used in this manuscript:

FTIR	Fourier transform infrared spectroscopy
LAS	linear amplitude sweep test
MSCR	multiple stress creep recovery test
MWAS	manufactured waste asphalt shingles
PAH	polycyclic aromatic hydrocarbons
PI	penetration index
RAS	reclaimed asphalt shingles
TOAS	tear-off asphalt shingles
